# Reliability, factor structure, and validity of the German version of the Trauma Symptom Checklist for Children in a sample of adolescents

**DOI:** 10.3402/ejpt.v6.27966

**Published:** 2015-10-23

**Authors:** Simone Matulis, Laura Loos, Nadine Langguth, Franziska Schreiber, Jana Gutermann, Caterina Gawrilow, Regina Steil

**Affiliations:** 1 Department of Clinical Psychology and Intervention, Institute of Psychology, Goethe University Frankfurt, Frankfurt, Germany; 2 German Institute for International Educational Research (DIPF), Frankfurt, Germany; 3 Center for Research on Individual Development and Adaptive Education of Children at Risk (IDeA-Center), Frankfurt, Germany

**Keywords:** Posttraumatic stress disorder, trauma, children, adolescents, self-report, assessment

## Abstract

**Background:**

The Trauma Symptom Checklist for Children (TSC-C) is the most widely used self-report scale to assess trauma-related symptoms in children and adolescents on six clinical scales. The purpose of the present study was to develop a German version of the TSC-C and to investigate its psychometric properties, such as factor structure, reliability, and validity, in a sample of German adolescents.

**Method:**

A normative sample of *N*=583 and a clinical sample of *N*=41 adolescents with a history of physical or sexual abuse aged between 13 and 21 years participated in the study.

**Results:**

The Confirmatory Factor Analysis on the six-factor model (*anger*, *anxiet*y, *depression*, *dissociation*, *posttraumatic stress*, and *sexual concerns* with the subdimensions *preoccupation* and *distress*) revealed acceptable to good fit statistics in the normative sample. One item had to be excluded from the German version of the TSC-C because the factor loading was too low. All clinical scales presented acceptable to good reliability, with Cronbach's α's ranging from .80 to .86 in the normative sample and from .72 to .87 in the clinical sample. Concurrent validity was also demonstrated by the high correlations between the TSC-C scales and instruments measuring similar psychopathology. TSC-C scores reliably differentiated between adolescents with trauma history and those without trauma history, indicating discriminative validity.

**Conclusions:**

In conclusion, the German version of the TSC-C is a reliable and valid instrument for assessing trauma-related symptoms on six different scales in adolescents aged between 13 and 21 years.

Worldwide, a high rate of youths have been exposed to traumatic events such as accidents, natural disasters, interpersonal violence, sexual abuse, or the violent death of close persons (e.g., Copeland, Keeler, Angold, & Costello, 2007). Epidemiological research reveals that traumatic events, especially physical or sexual abuse in childhood, have severe impacts on psychological and psychosocial functioning in youths (Fergusson, Boden, & Horwood, 2008). Sequelae include symptoms of posttraumatic stress, depression, anxiety, general emotional dysregulation, anger, dissociation, behavioral problems, and—primarily in survivors of sexual abuse—sexual problems, that is, sexual thoughts or feelings that occur earlier or more frequently than expected, sexual conflicts, negative responses to sexual stimuli, and fear of being sexually exploited (Boney-McCoy & Finkelhor, 1995; D'Andrea, Ford, Stolbach, Spinazzola, & van der Kolk, 2012; Guterman, Cameron, & Hahm, 2003; Silverman, Reinherz, & Giaconia, 1996). Although posttraumatic stress disorder (PTSD) frequently follows trauma (e.g., Giaconia et al., 1995), the effects of multiple and interpersonal trauma are often complex and extend beyond core PTSD symptoms, that is, they are associated with symptoms of dissociation, emotion regulation difficulties, suicidal ideation, or aggressive behavior (Brière, Kaltman, & Green, 2008).

Various self-report instruments have been developed to measure specific trauma-related symptoms in children and adolescents such as the University of California Los Angeles PTSD Reaction Index (UCLA-PTSD-RI; Steinberg, 2004), the Adolescent Dissociative Experiences Scale (A-DES; Armstrong, Putnam, Carlson, Libero, & Smith, 1997), or the Child Sexual Behavior Inventory (Friedrich et al., 1992). In contrast to the aforementioned measures focusing on specific reported trauma-related symptoms, the Trauma Symptom Checklist for Children (TSC-C) developed by John Brière (1996) is a broad-based, multi-dimensional self-report questionnaire that directly examines trauma-related symptoms. It comprises 54 items covering six clinical scales (anger, anxiety, depression, dissociation, depression, and sexual concerns). Every clinical scale includes 9–10 items, although some items apply to more than one clinical scale. The TSC-C is suitable for assessing different clusters of trauma-related symptoms and provides an individual profile of the child's symptoms. It is used in a large body of studies on posttraumatic reactions in children and adolescents aged between 8 and 18 years (e.g., Kolko et al., 2010; Nilsson, Gustafsson, & Svedin, 2012) and in clinical trials for the treatment of children and adolescents who have been abused (e.g., Cohen, Mannarino, & Knudsen, 2005; Lanktree & Brière, 1995a).

The TSC-C is the most widely used self-report scale for measuring trauma-related symptoms in youth (Balaban, 2006). The psychometric properties of the TSC-C have been studied in clinical and non-clinical samples of individuals between 8 and 17 years old (Crouch, Smith, Ezzell, & Saunders, 1999; Lanktree et al., 2008; Sadowski & Friedrich, 2000; Singer, Anglin, Song, & Lunghofer, 1995; see Supplementary file for detailed information). The results confirm that the TSC-C is a reliable and valid instrument to measure trauma-related symptoms in children and adolescents. The TSC-C has been used and its psychometric properties have been assessed in several languages and in countries such as the US, Iran (Mohammadkhani, Nazari, Dogaheh, Mohammadi, & Azadmehr, 2007), Sweden (Nilsson, Wadsby, & Svedin, 2008), the Netherlands (Bal & Uvin, 2009), China (Li et al., 2009), and Korea (Chung, 2014). Although Nilsson et al. (2008) and Chung (2014) conducted exploratory factor analyses to test the TSC-C's factorial validity, the other studies only focused on reliability and validity aspects. Both workgroups came to similar findings: after performing factor analysis using principal component analysis and varimax rotation with Kaiser normalization, they confirmed a six-factor model that explained more than 50% of the variance.

In summary, the TSC-C is useful for both research and practice because of its ability to assess different trauma-related symptoms, its robust psychometric properties, and its frequent international use in research. However, thus far, a German version of the TSC-C is lacking. For this reason, we developed a German version of the TSC-C (German TSC-C) and evaluated its use in adolescents. Because in the German healthcare system, child and adolescent therapists treat patients up to the age of 21 years, we chose to study the psychometric properties in adolescents aged up to 21 years. We sought to examine the instrument's psychometric properties, such as reliability and validity, in both a large non-clinical German adolescent sample and a clinical sample (CS) of adolescents with a history of physical or sexual abuse. Furthermore, we aimed to examine whether the German TSC-C shows the same factor structure as the original.

Therefore, we investigated the following research questions:

1. Does the German TSC-C exhibit the same six-factor structure (*anxiety*, *anger*, *depression*, *dissociation*, *posttraumatic stress*, and *sexual concerns*) as Brière's original TSC-C?
2. How good is the internal consistency in each of those six clinical scales?
3. Do the clinical scales of the German TSC-C correlate highly with measurements assessing similar psychopathology (convergent validity)?
4. Does the German TSC-C discriminate well between those participants who had experienced a trauma and those who had not?

## Method

To investigate the validity and reliability of the German TSC-C, we investigated a non-clinical normative sample (NS) and a CS of adolescent survivors of physical or sexual abuse.

### Recruitment and procedure

The current investigation in the NS was part of a larger study (Langguth et al., in press). Students aged between 13 and 21 with no physical impairment and with sufficient knowledge of the German language were included. The recruitment of the NS is described in more detail in Langguth et al. (2015).

Participants in the CS were recruited at a specialized PTSD outpatient center. Patients were assessed to participate in studies with the aim of evaluating a specialized treatment for adolescents with PTSD after sexual or physical abuse: Developmentally Adapted Cognitive Processing Therapy for adolescents with PTSD after sexual or physical abuse (Matulis, Resick, Rosner, & Steil, 2014). After initial contact with the adolescent patient or caregivers, patients were screened for inclusion and exclusion criteria. Adolescents and youth aged between 13 and 21 who reported physical or sexual abuse were included. The exclusion criteria are described in detail in Matulis et al. (2014). Informed consent was provided by the patient and, if the adolescent was under 18 years of age, by the patient's caregivers. Both studies were approved by the local ethics committee.

### Participants

*Normative sample*. The NS comprises a total (*N*) of 583 participants. All participants attended public secondary schools (grades 8–13) in a large urban area in Germany. In total, 247 participants (42.4%) had immigrant background. Further sample characteristics are presented in Table 1.

**Table 1:** Demographics of the normative and clinical adolescent samples

Demographic variables	Results
Normative sample (*N*=583)	
Age *M*, SD Range	17.44, 1.98 13–21
Sex Females (*n*, %)	185, 31.7
History of trauma according to DSM-IV (trauma subsample) (*n*, %) Physical assault (*n*, %) Sudden death of a loved one (*n*, %) Severe accident (*n*, %) Contact with a dead body (*n*, %) Painful medical treatment (*n*, %) Sexual assault (*n*, %)	260, 44.6 170, 65.4 121, 46.5 97, 37.3 85, 33.6 72, 27.7 47, 18.1
Clinical sample (*N*=41)	
Age *M*, SD Range	17.61, 1.97 14–21
Sex Females (*n*, %)	32, 78
History of trauma (*n*, %) Sexual abuse (*n*, %) Physical abuse (*n*, %) Further traumatization (*n*, %)	41, 100 33, 80.5 32, 78 22, 53.7
PTSD Diagnosis according to DSM-IV (*n*, %) Score (*M*, SD)	34, 82.9 59.66, 26.49
Comorbid disorders according to DSM-IV (*M*, SD)	2.56, 1.40
Affective disorders (*n*, %) Anxiety disorders (*n*, %) Substance abuse/dependency (*n*, %) Eating disorders (*n*, %) Somatoform disorders (*n*, %) Conduct disorder (*n*, %)	24, 58.54 19, 46.34 7, 17.07 5, 12.20 4, 9.76 3, 7.32

PTSD diagnosis and score were assessed using the Clinician-Administered PTSD Scale (CAPS; Blake et al., 2000); comorbid diagnoses were assessed using the Structured Clinical Interview for DSM-IV (SCID-I; First, Spitzer, Gibbon, & Williams, 1997) and the Diagnostic Interview for Mental Disorders in Childhood and Adolescence (Kinder-DIPS; Schneider, Unnewehr, & Margraf, 2009).

*Clinical sample*. The CS consists of 41 adolescents. All participants had a history of physical or sexual abuse (Table 1).

### Measures

*Trauma Symptom Checklist for Children (TSC-C; Brière, 1996)*. The TSC-C is a self-report instrument for the assessment of psychological impairments following traumatization. It has 54 items and requires approximately 15 min to complete. The TSC-C yields two validity scales, Hyper-response and Under-response (UND), and six clinical scales that cover a broad range of possible groups of trauma-related symptoms: *Anger* (ANG), *Anxiety* (ANX), *Depression* (DEP), *Dissociation* (DIS), *Posttraumatic Stress* (PTS), and *Sexual Concerns* (SC). Questions are answered on a four-point Likert scale (0 “never,” 1 “sometimes,” 2 “lots of times,” and 3 “almost all of the time”).

The TSC-C (Brière, 1996) was studied in a NS of *N*=3,008 children and adolescents. All clinical scales exhibited good reliability with a mean Cronbach's α across the six clinical scales of .84. Furthermore, the reliability of the TSC-C was also studied in three different samples that had been recruited from child abuse centers (Elliott & Brière, 1994: *n*=399; Lanktree & Brière, 1995b: *n*=105; Nelson-Gardell, 1995: *n*=103) with good Cronbach's α (α=.81, .86, and .85, respectively). Further studies confirmed the TSC-C to be valid in CSs (e.g., Lanktree et al., 2008; Sadowski & Friedrich, 2000).

For the German TSC-C, all items were translated into German and translated back by a native speaker in the original language (back translation). There was high concordance between the translated and back-translated versions. In his original study, Brière allowed several items (11, 24, and 25) to load on more than one factor. To reduce content overlap between the factors, we elected to assign each item to only one factor. Item 11 refers in Brière's study to both the PTS and DIS scales, which is understandable, as this item contains two different statements. To keep this item as simple and comprehensible as possible, we decided to translate only one part of this item (“Trying not to think.”) and assigned it to the PTS scale. Items 24 and 25 refer in Brière's study to the ANX and PTS scales. We decided to account for these items in the ANX scale because, in our opinion, the wording and content of the German translation are more related to this scale.

*Adolescent Dissociative Experiences Scale (Armstrong et al., 1997; in German, HDI; Brunner, Resch, Parzer, & Koch, 2008)*. The A-DES is a 30-item self-report measure assessing how often adolescents aged 10–21 years old actually experience dissociative symptoms. The adolescent responds to statements on an 11-point scale (ranging from 0 “never” to 10 “always”). The instrument exhibits good reliability and validity (Armstrong et al., 1997). The German HDI also shows good reliability (Cronbach's α=.94; Brunner et al., 2008). The A-DES was used to test concurrent validity in the CS (i.e., based on the correlation between the A-DES and the DIS). Cronbach's α was .94 in the present sample.

*Beck Depression Inventory (BDI-II; Beck, Steer, & Brown*, *1996*; *in German, Hautzinger, Keller, & Kühner*, *2006**)*. The BDI-II is the most commonly used questionnaire for assessing depressive symptoms during the past 2 weeks in adults and adolescents over the age of 13. The items are answered on a four-point Likert scale. The German version of the BDI-II shows good reliability and validity in clinical and non-clinical samples (Cronbach's α≥.84; Kühner, Bürger, Keller, & Hautzinger, 2007). In the present study, the BDI-II was used to investigate concurrent validity within the CS (i.e., based on the correlation between the BDI-II and DEP). Cronbach's α was .94.

*Center for Epidemiological Studies—Depression Scale (CES-D; Radloff*, *1977*; *in German, Hautzinger, Bailer, Hofmeister, & Keller, 2012)*. The CES-D is a valid and reliable questionnaire that measures depressive symptoms during the past 4 weeks. All questions are answered using a four-point Likert scale (0 “rarely,” 1 “sometimes,” 2 “often,” and 3, “mostly”). The German version shows good reliability in adults (Cronbach's α ranging from .89 to .92) as well as in children and adolescents over the age of 12 years (Cronbach's α ranging from .82 to .88; Hautzinger et al., 2012). It was used in the current study to test concurrent validity in the NS (correlation between the CES-D depression scale and DEP). In the present study, the CES-D exhibited good internal consistency (Cronbach's α=.88).

*Depression Inventory for Children and Adolescents (DICA; Stiensmeier-Pelster, Schürmann, & Duda, 2000)*. The DICA is the German version of the Children's Depression Inventory (Kovacs, 1985). It evaluates the depressive symptoms of 8- to 16-year-old children and adolescents using three-point Likert scale questions. The internal consistency (Cronbach's α=.87) and the convergent and discriminant validity can be classified as good (Stiensmeier-Pelster et al., 2000). The DICA was used in a subsample of the CS to investigate the concurrent validity (i.e., the correlation between the DICA and DEP). In the present study, Cronbach's α was .61.

*State–Trait–Anxiety–Depression Inventory (STADI; Laux, Hock, Bergner-Köther, Hodapp, & Renner, 2012)*. The STADI is a self-report questionnaire that examines anxiety and depression in individuals aged 16 years and older. “State” refers to actual symptoms, and “Trait” refers to general experiences. Every item is answered using a four-point Likert scale. The reliability and validity of the STADI have been confirmed (e.g., Trait Anxiety Cronbach's α=.88; Laux et al., 2012). The STADI was used to test concurrent validity in the NS (i.e., the correlation between the STADI-Trait Anxiety subscale and ANX). In this sample, Cronbach's α was .87.

UCLA-PTSD-RI PTSD Reaction Index for DSM-IV (UCLA-PTSD-RI; Steinberg, Brymer, Decker, & Pynoos, 2004; in German, Ruf, Schauer, & Elbert, 2011). The UCLA-PTSD-RI PTSD Reaction Index for DSM-IV (Revision 1) is used to assess trauma exposure and posttraumatic stress symptoms among children and adolescents. It consists of a brief lifetime trauma screening, an evaluation of A1 and A2 DSM-IV criteria, and 22 items assessing posttraumatic stress symptoms during the past month. The adolescent version is intended for youth aged 13 years or older (responses range from 0, “none of the time”, to 4, “most of the time”). Several studies have demonstrated the high reliability and validity of the UCLA-PTSD-RI index and good to excellent internal consistency (α=.88–.91; Steinberg et al., 2013). In the current study, the UCLA-PTSD-RI was used in both samples to study the frequency of trauma and to test concurrent validity (i.e., the correlation between the UCLA-PTSD-RI score and PTS). Internal consistencies were good in both samples (NS: Cronbach's α=.92; CS: Cronbach's α=.87).”

### Statistical analysis

Missing data analyses were computed according to Tabachnick and Fidell (2007). In the NS, 1.29% of the data were missing. Missing data were imputed using the multiple imputation technique because the data were not missing completely at random, as indicated by the results of Little's Missing-Completely-At-Random Test (MCAR test; e.g., Little & Rubin, 1989; Little's MCAR *χ*^2^(12,651)=15,868.678, *p*<.001). The multiple data sets derived from the multiple imputation technique were used to conduct all analyses in the normative group. In the CS, .63% of the data were missing. Because we found that the missing data in the CS were missing completely at random (Little's MCAR *χ*^2^(2,960)=.00, *p*=1.000), we estimated values for the missing data using an expectation–maximization method.

Research question 1: To evaluate the factor structure of the German TSC-C in the NS, we conducted a Confirmatory Factor Analysis (CFA) in Mplus Version 6 (Muthén & Muthén, 1996–2010). We used the following indices to evaluate the fit of the six-factor model: *χ*^2^, the Bayesian Information Criterion (Schwarz, 1978), the Comparative Fit Index (CFI; Bentler, 1990), the Tucker Lewis Index (TLI), the Root Mean Square Error of Approximation (RMSEA; Steiger, 1990), and the Standardized Root Mean Square Residual (SRMR; Bentler, 1995). Suggestions for interpretation are displayed in Table 2. Furthermore, we compared a six-factor model to a one-factor model, considering the fit indices. Because the present sample does not follow a multivariate normal distribution, we used robust maximum likelihood estimation with robust standard errors. To optimize the German TSC-C for the German adolescent population, we excluded items with a factor loading *λ*<.30 (Hair, Anderson, Tatham, & Black, 1995).

**Table 2:** Fit statistics of the German version of the TSC-C by model

	*χ* ^2^ (df)	BIC	CFI	TLI	RMSEA	SRMR
Six-factor model	2,660.78 (1,303)	60,868.04	.85	.84	.04	.06
One-factor model	4,507.46 (1,325)	63,196.42	.65	.63	.06	.08

BIC, Bayesian information criterion; CFI, comparative fit index; RMSEA, root mean square error of approximation; SRMR, standardized root mean square residual; ANG, anger; ANX, anxiety; DEP, depression; DIS, dissociation; PTS, posttraumatic stress; SC, sexual concerns. Suggestions for interpreting the index as acceptable, according to Schermelleh-Engel, Moosbrugger, and Müller (2003): *χ*^2^/df≤3; CFI≥.95; TLI≥.95 RMSEA≤.08; SRMR≤.10.

Research question 2: We evaluated the reliability of each clinical scale using Cronbach's α. To enhance the comparability of the present study's findings to those of previous studies that mainly investigated children and adolescents up to 18 years, we conducted reliability analyses in a younger (13–18 years) and an older subsample (19–21 years) of the NS.

Research question 3: To examine the concurrent validity of the TSC-C, we computed two-way product–moment correlations between the TSC-C scales and the corresponding questionnaires. Because the CS was recruited in different psychotherapy studies, two different depression measures were used (BDI-II and DICA). To investigate the concurrent validity of the TSC-C DEP, we used a combined depression score consisting of the *z*-standardized BDI-II and DICA scores in the CS.

Research question 4: We computed a MANOVA to test for differences in the TSC-C scores between the subsample of the NS with no self-reported trauma history, the subsample with self-reported trauma history, and the CS. The significance level was set at *p*<.05 (two-tailed). *Post hoc* analyses (ANOVAs) were performed to test for group differences on each of the six clinical scales. Finally, a series of *t*-tests were computed to test for group differences on each clinical scale. Effect sizes (Cohen's *d*) are reported. A Bonferroni adjustment was applied for the *post hoc* analyses to avoid an excess of type I errors. We only report the corrected *p* values. Because prior studies found differences in TSC-C scores depending on age and sex (e.g., Nilsson et al., 2008), we also tested whether the groups differed in age or sex in the present study using ANOVA and the chi-square test of independence. The statistics were calculated using SPSS^®^ Version 22.0.

## Results

### Research question 1: factor analysis

The results of the CFA in the NS are displayed in Table 2. It shows the fit statistics for the six-factor model and a one-factor model. Of the 54 items, 50 items show factor loadings with *λ*≥.30 (Table 3). Item 26 (DEP scale) showed a factor loading of *λ*=.24. Therefore, we excluded this item from further analysis. On the SC scale, three items (34, 40, and 54) show low factor loadings (*λ*_34_=.19, *λ*_40_=.28, *λ*_54_=.14). In Brière's original version of the TSC-C, these items refer to the SC subscale sexual distress. Therefore, using the methods described in Brière (1996), we computed a two-factor model for the SC scale in which the sub-dimension Sexual Preoccupation (SC-P) comprises seven items and the subscale Sexual Distress (SC-D) comprises the aforementioned three items. This model shows good fit statistics (CFI: .96, TLI: .94, RMSEA: .05, SRMR: .04). All items of the SC-P and SC-D subscales show factor loadings *λ*≥.30.

**Table 3:** Factor loadings of the six-factor model of the German version of the TSC-C

TSC-C item	Factor	Factor loading (*λ*)
1. Bad dreams or nightmares	PTS	.46
2. Feeling afraid something bad might happened	ANX	.59
3. Scary ideas or pictures just pop into my head	PTS	.64
4. Wanting to say dirty words	SC-P	.42
5. Pretending I am someone else	DIS	.51
6. Arguing too much	ANG	.45
7. Feeling lonely	DEP	.73
8. Touching my private parts too much	SC-P	.63
9. Feeling sad or unhappy	DEP	.74
10. Remembering things that happened that I didn't like	PTS	.74
11. Going away in my mind, trying not to think	PTS	.51
12. Remembering scary things	PTS	.77
13. Wanting to yell and break things	ANG	.68
14. Crying	DEP	.52
15. Getting scared all of a sudden and don't know why	ANX	.68
16. Getting mad and can't calm down	ANG	.67
17. Thinking about having sex	SC-P	.76
18. Feeling dizzy	DIS	.42
19. Wanting to yell at people	ANG	.75
20. Wanting to hurt myself	DEP	.59
21. Wanting to hurt other people	ANG	.53
22. Thinking about touching other people's private parts	SC-P	.72
23. Thinking about sex when I don't want to	SC-P	.60
24. Feeling scared of men	ANX	.37
25. Feeling scared of women	ANX	.30
26. Washing myself because I feel dirty on the inside^a^	DEP	
27. Feeling stupid or bad	DEP	.72
28. Feeling like I did something wrong	DEP	.61
29. Feeling like things aren't real	DIS	.73
30. Forgetting things, can't remember things	DIS	.54
31. Feeling like I am not in my body	DIS	.67
32. Feeling nervous or jumpy inside	ANX	.66
33. Feeling afraid	ANX	.84
34. Not trusting because they might want sex	SC-D	.53
35. Can't stop thinking about something bad that happened to me	PTS	.71
36. Getting into fights	ANG	.36
37. Feeling mean	ANG	.49
38. Pretending I am somewhere else	DIS	.56
39. Being afraid of the dark	ANX	.46
40. Getting scared or upset when I think about sex	SC-D	.67
41. Worrying about things	ANX	.51
42. Feeling like nobody likes me	DEP	.66
43. Remembering things I don't want to remember	PTS	.76
44. Having sex feelings in my body	SC-P	.70
45. My mind going empty or blank	DIS	.59
46. Feeling like I hate people	ANG	.51
47. Can't stop thinking about sex	SC-P	.77
48. Trying not to have any feelings	DIS	.58
49. Feeling mad	ANG	.54
50. Feeling afraid somebody will kill me	ANX	.47
51. Wishing bad things had never happened	PTS	.64
52. Wanting to kill myself	DEP	.54
53. Daydreaming	DIS	.45
54. Getting upset when people talk about sex	SC-D	.44

ANG, anger; ANX, anxiety; DEP, depression; DIS, dissociation; PTS, posttraumatic stress; SC-D, sexual distress; SC-P, sexual preoccupation.

a Removed from final model based on factor loading<.30.

Finally, the six-factor model comprising the six clinical scales with SC representing a higher-order factor consisting of SC-P and SC-D shows fit indices that can be interpreted as acceptable (SRMR, *χ*^2^/df) or good (RMSEA). The CFI and TLI values are slightly smaller than the recommended values for acceptable fits. Compared with a one-factor model, the six-factor model shows better fit indices (Table 2).

All correlations among the clinical scales were positive and significant with correlations ranging from *r*=.14 (ANX—SC-P) to *r*=.97 (SC—SC-P) in the NG and *r*=.19 (SC-D—SC-P) to *r*=.81 (SC—SC-P) in the CG (Table 4).

**Table 4:** Correlations between the TSC-C Clinical Scales and the total TSC-C Scale in the normative and clinical samples

	TSC-C ANG	TSC-C ANX	TSC-C DEP	TSC-C DIS	TSC-C PTS	TSC-C SC	SC-P
TSC-C ANG							
TSC-C ANX	.52 (.52)^a^						
	.62 (.54)^b^						
TSC-C DEP	.57 (.56)^a^	.72 (.76)^a^					
	.63 (.62)^b^	.72 (.75)^b^					
TSC-C DIS	.62 (.61)^a^	.68 (.69)^a^	.71 (.70)^a^				
	67 (.70)^b^	.52 (.66)^b^	.77 (.77)^b^				
TSC-C PTS	.55 (.73)^a^	.70 (.81)^a^	.70 (.73)^a^	.64 (.74)^a^			
	.46 (.57)^b^	.78 (.86)^b^	.63 (.73)^b^	.51 (.70)^b^			
TSC-C SC	.39 (.37)^a^	.23 (.45)^a^	.24 (.41)^a^	.32 (.51)^a^	.30 (.51)^a^		
	.51 (.50)^b^	.58 (.32)^b^	.45 (.38)^b^	.36 (.57)^b^	.39 (.51)^b^		
SC-P	.36 (.40)^a^	.14 (.32)^a^	.16 (.34)^a^	.25 (.44)^a^	.22 (.41)^a^	.97 (.92)^a^	
	.45 (.43)^b^	.36 (.32)^b^	.31 (.38)^b^	.26 (.45)^b^	.21 (.35)^b^	.81 (.91)^b^	
SC-D	.24 (.19)^a^	.39 (.48)^a^	.35 (.37)^a^	.34 (.41)^a^	.35 (.47)^a^	.47 (.72)^a^	.22 (.40)^a^
	.34 (.42)^b^	.56 (.66)^b^	.39 (.52)^b^	.30 (.53)^b^	.41 (.56)^b^	.74 (.74)^b^	.19 (.39)^b^

ANG, anger; ANX, anxiety; DEP, depression; DIS, dissociation; PTS, posttraumatic stress; SC, sexual concerns. Pearson's correlation sign two-tailed *p*<.05 for all calculations.

a Correlation in NS [correlations in Brière's (1996) standardization sample; correlations between clinical scales and the total scale were not reported].

b Correlation in CS [correlations in Sadowski and Friedrich's (2000) psychiatric adolescent sample].

### Research question 2: reliability

Table 5 reports the internal consistencies for each of the TSC-C scales. In the NS, analyses were conducted for a younger sample (13–18 years) and an older sample (19–21 years). The overall Cronbach's α was .94. For the complete NS, the internal consistencies of the clinical scales varied between Cronbach's α=.80 (ANG scale) and α=.86 (DEP scale). Compared with the younger subsample, the values for the older subsample were higher. For the CS, the values varied between α=.72 (SC scale) and α=.87 (DEP scale).

**Table 5:** Cronbach's α's for the TSC-C Subscales and the TSC-C Total Scale in the normative and clinical samples

	Normative sample		
		
Clinical scale	13–18 years	19–21 years	Clinical sample
TSC-C ANG	.78 (.89)	.84	.79 (.87)
TSC-C ANX	.79 (.82)	.84	.84 (.83)
TSC-C DEP	.85 (.86)	.88	.87 (.85)
TSC-C DIS	.79 (.83)	.80	.80 (.80)
TSC-C PTS	.85 (.87)	.85	.86 (.85)
TSC-C SC	.81 (.77)	.79	.74 (.67)
SC-P	.84 (.81)	.83	.72
SC-D	.45 (.64)	.68	.86
TSC-C total scale	.94		.94
UND	.82 (.85)	.85	.85
HYP	.61 (.66)	.70	.61

The results from Brière's (1996) study (*N*=3,008) are displayed in parentheses in the second column; the results from the study conducted in a child abuse center (Elliott & Brière, 1994; *N*=399) are shown in parentheses in the third column.

ANG, anger; ANX, anxiety; DEP, depression; DIS, dissociation; PTS, posttraumatic stress; SC, sexual concerns; UND, validity scale under-response; HYP, validity scale hyper-response.

### Research question 3: concurrent validity

Concurrent validity was established by two-way product-moment correlations between the TSC-C scales and further measurements that were completed in the NS (CES-D, STADI, and UCLA-PTSD-RI) and the CS (A-DES, DICA, BDI-II, and UCLA-PTSD-RI). In the NS, we observed high correlations between DEP and the CES-D (*r*=.72, *p*<.001), between ANX and the STADI (*r*=.60, *p*<.001), and between PTS and the UCLA-PTSD-RI (*r*=.76, *p*<.001). In the CS, we observed high correlations between DIS and the A-DES (*r*=.71, *p*<.001), between DEP and the *z*-standardized depression score (*r*=.81, *p*<.001), and between PTS and the UCLA-PTSD-RI (*r*=.79, *p*<.001) (for full information on all correlations see Supplementary file).

### Research question 4: discriminative validity

To test discriminative validity, differences in the TSC-C scores between the subsample of the NS with a history of trauma, the subsample of the NS without a history of trauma, and the CS were investigated. An analysis of variance showed that the groups differed significantly in age (*F*(2,621)=11.91, *p*<.001). *Post hoc* analyses using the Scheffe' *post hoc* criterion for significance (Scheffé, 1953) indicated that the traumatized subsample of the NS was significantly older than the non-traumatized subsample (Δ*M*=.79; *p*<.001). A chi-square test of independence was performed to examine the relationship between sex and group. The relationship between these variables was significant (*χ*^2^ (2, *N*=604)=40.14, *p*<.001) in that that there were relatively more girls in the traumatized subsample than in the non-traumatized subsample of the NS. Thus, we included age and sex as covariates in the further analysis.

Wilks's statistic indicated that there was a significant effect of group on the TSC-C scores after controlling for age and sex, Λ=.68, *F* (14, 1,188)=18.15, *p*<.001, *η*^2^=.18. Table 6 shows the detailed results of the MANCOVA with the results for each of the clinical scales.

**Table 6:** MANCOVA for the TSC-C differences between the normative and the clinical samples, with age and gender as covariates

	NS1		NS2		CS		Group				
									NS2-NS1	CS-NS1	CS-NS2
Clinical scale	*M*	*SD*	*M*	*SD*	*M*	*SD*	*F*	df	*d*	*d*	*d*
TSC-C ANG	5.14	3.93	7.69	4.66	7.95	4.81	20.69***	2	NS2>NS1*** .60	CS>NS1*** .70	CS>NS2 ns
TSC-C ANX	3.57	3.36	5.77	4.10	11.33	5.74	53.20***	2	NS2>NS1*** .59	CS>NS1*** 2.10	CS>NS2*** 1.28
TSC-C DEP	3.84	3.71	6.14	4.61	11.19	5.87	38.72***	2	NS2>NS1*** .56	CS>NS1*** 1.84	CS>NS2*** 1.05
TSC-C DIS	4.18	3.86	6.49	4.38	7.83	5.38	15.66***	2	NS2>NS1*** .56	CS>NS1*** .90	CS>NS2 ns
TSC-C PTS	4.52	3.73	8.50	4.97	14.61	5.44	92.62***	2	NS2>NS1*** .92	CS>NS1*** 2.55	CS>NS2*** 1.21
TSC-C SC	5.01	4.18	6.61	5.04	5.73	4.59	7.81***	2	NS2>NS1^**^ .35	CS>NS1 ns	CS<NS2 ns
SC-P	4.51	3.87	5.72	4.50	3.60	3.17	5.99^**^	2	NS2>NS1^**^ .30	CS<NS1 ns	CS<NS2^**^ .49
SC-D	.50	1.04	.89	1.45	2.16	2.77	19.43***	2	NS2>NS1*** .32	CS>NS1*** 1.23	CS>NS2* .75

NS1, non-trauma subsample of the normative sample; NS2, trauma subsample of the normative sample; CS, clinical sample; M, mean value; *SD*, standard deviation; *d*, Cohen's *d*; ANG, anger; ANX, anxiety; DEP, depression; DIS, dissociation; PTS, posttraumatic stress; SC, sexual concerns.

* *p<*.01;

*** *p<*.001; ns, not significant.

*Post hoc* tests revealed that in the NS, the traumatized subsample had higher scores on the TSC-C and all clinical scales than the subsample with no self-reported trauma history; the effects were small (SC-P: *d*=.30) to large (PTS: *d*=.92; Table 6). The CS presented significantly higher scores than the NS subsample with no self-reported trauma for all scales except the SC scale, with medium (ANG: *d*=.70) to large (PTS: *d*=2.55) effects. Considering the subscales of the SC scale, the analyses show that the CS had higher scores on the SC-P scale (*d*=1.23) but not on the SC-D scale. Compared with the traumatized subsample of the NS, the CS showed higher scores on the ANX, DEP, and PTS scales, with large effects. Considering the SC subscales, the CS reported lower scores on the SC-P scale but higher scores on the SC-D scale.

## Discussion

At present, there is no German measurement that assesses the broad range of trauma-related symptoms in children and adolescents. Therefore, we developed a German version of the TSC-C (Brière, 1996), and evaluated the German TSC-C in a sample of adolescents between 13 and 21 years old. This is the first study on the German version of the TSC-C using both a large normative and a CS sample. In line with our hypothesis derived from the original version of the TSC-C, we observed a six-factor structure, with every scale demonstrating acceptable to good Cronbach's αs, high correlations of the TSC-C scales with similar measures (concurrent validity), and TSC-C scores that reliably differentiated between traumatized and non-traumatized participants.

The overall scale exhibited excellent internal consistencies for the NS and the CS in accordance with results reported in a Swedish study on the TSC-C (Nilsson et al., 2008). In the NS, the reliability analyses demonstrated good reliability for all six clinical scales, whereas the values for the older age group were higher than those of the younger age group on all scales except the SC scale. When comparing the results for the younger age group with the results obtained by Brière (1996), Bal, Crombez, Van Oost, and Debourdeaudhuij (2003), or Chung (2014), who investigated psychometric properties in youths up to the age of 18, the results are comparable. The SC subscale SC-D showed the lowest value in the NS. This result is also in line with previous studies: for example, Nilsson et al. (2008) report a Cronbach's α of .54 on the SC-D scale. For the CS, all clinical scales presented acceptable to good reliability. This is in line with Crouch et al. (1999), who studied the TSC-C in a sample of *N*=80 sexually abused children and adolescents, and Sadowski and Friedrich (2000), who investigated the psychometric properties of the TSC-C in a psychiatric adolescent sample (*N*=119).

Previous studies of the TSC-C report lower internal consistencies for the SC scale (Brière, 1996; Chung, 2014; Crouch et al., 1999; Nilsson et al., 2008). One might speculate that these relatively low values in the previous studies could be explained by the fact that Brière originally proposed the SC scale to have two subscales, namely *Sexual Preoccupation* and *Sexual Distress*, thus resulting in a larger inconsistency. However, in the present study, three of the four items that were supposed to load on the *Sexual Distress* subscale had to be excluded because they presented low loadings on the SC scale. This raised the SC scale's internal consistency.

In the present study, we decided to exclude items that presented excessively low factor loadings (*λ*<.30) from the German TSC-C. Item 26 did not sufficiently load on the originally proposed DEP scale. One might assume that this item reflects more of a *feeling of being contaminated* than an aspect of depression. As victims of sexual violence often suffer from the feeling of being contaminated (Jung & Steil, 2013; Steil, Jung, & Stangier, 2011), we supposed that this item might be related to the PTS scale. However, when computing a PTS factor model including item 26, this item still revealed a factor loading <.30. Thus, we excluded this item from the German TSC-C.

Using CFA, we were able to find a factor structure close to that of Brière's original TSC-C version (Brière, 1996). The high concordance between Brière's original study and the results of the German version of the TSC-C is remarkable considering the broad structure of the instrument and the differences concerning the structure of the studied samples. First, Brière's standardization sample has a higher share of girls (53 vs. 35% in the present NS). Second, contrary to our study, Brière's sample also covers children under the age of 13 but does not refer to adolescents between 18 and 21 years of age. Moreover, we observed lower means on five of the six clinical scales (not on the SC scale). The same pattern was found in the Swedish and Korean investigations. The lower scores of the present study's samples could be explained by the fact that the adolescents in the present study's NS were more likely to underreport symptoms than were the older participants in Brière study, as indicated by the higher underreporting scale scores of the present study's normative group (UND score in the present sample: males: *M*=7.56, SD=4.54; females: *M*=10.59, SD=4.56 vs. UND score in Brière's standardization sample: males: *M*=2.9, SD=2.6; females: *M*=1.7, SD=2.0).

To investigate aspects of validity, we studied the concurrent and discriminative validity of the German TSC-C. As hypothesized, in the NS, we found high and significant correlations between the ANX scale and the STADI, between the DEP scale and the CES-D, and between the PTS scale and the UCLA-PTSD-RI. Although other studies on the TSC-C have also considered concurrent validity in their NSs (Brière, 1996; Chung, 2014), they are not directly comparable to our results because they used different instruments. In line with the results in our NS, as hypothesized, we also found high and significant correlations between the DIS scale and the A-DES, between the DEP-scale and the DICA and the BDI-II, and between the PTS scale and the UCLA-PTSD-RI. These results are also comparable to those reported by Sadowski and Friedrich (2000), who observed a correlation of *r*=.81 between the DEP scale and the BDI and *r*=.79 between the DIS scale and the A-DES. These findings indicate that concurrent validity for the German TSC-C is confirmed.

Further support for the validity of the German TSC-C is based on the finding that in the NS, the adolescents with a trauma history had significantly higher scores on all clinical scales compared with the adolescents without a trauma history, whereas the participants in the CS had higher scores on five of the six clinical scales compared with the non-traumatized subsample of the NS after controlling for age and sex differences. Considering both of the SC subscales, the participants in the CS appeared to report more symptoms of sexual distress than those in the NS. At the same time, the CS participants reported as many symptoms of SC-P as the non-traumatized subsample of the CS and even fewer symptoms than the traumatized subsample of the NS did. These findings are in line with those reported by Nilsson et al. (2008), who also used normative and CSs. Considering that the CS in the present study comprised a high proportion of victims of sexual violence, one might speculate that symptoms of sexual distress—such as “not trusting other people because they might want sex” or “getting upset when people talk about sex”—might be a symptom group that is characteristic of youths who have experienced severe interpersonal violence. However, to prove this hypothesis, future research must examine and compare the psychometric properties of the TSC-C—especially the SC scale—in samples that report having experienced different types of trauma.

## Limitations and implications for future research

Several limitations and weaknesses may impede the generalizability of our results. First, participants in the NS were allocated to the trauma subsample if they reported a traumatic event according to the DSM-IV in the UCLA-PTSD-RI. Measuring traumatic events through the sole use of questionnaires is much less valid than doing so through clinical interviews. Participants might have misunderstood the questions and given false positive answers here. However, assessing traumatic events via a clinical interview is very difficult if a large sample is needed. Second, our NS is not representative of the population of German adolescents with respect to age, sex, and educational level. Contrary to the originally intended and recommended use of the TSC-C in children from 8 to 16 years of age, the German TSC-C was studied here in a NS with a mean age of approximately 17 years, whereas 52.1% of participants in the NS of the present study were between 18 and 21 years old. Our results—from the first study administering the TSC-C to adolescents older than 17 years—imply that the instrument is appropriate for application in this age group. However, the extent to which the results of the present study on the German TSC-C are generalizable to younger samples remains unclear. However, in Brière's original standardization sample, 70% of the participants were between 15 and 16 years old. Further investigations of the psychometric properties of the German TSC-C in children below the age of 13 are needed. With regard to sex, our NS predominantly comprised male participants. Concerning educational level, in our NS, higher educational levels were underrepresented compared with the general population of 13- to 21-year-old Germans. All of this should be considered in future research on the German TSC-C.

Finally, in our NS, participants with a trauma history were over-represented compared with the results of older epidemiological studies using German samples. Although 44.6% of our NS reported having experienced one or more traumas, Perkonigg, Kessler, Storz, and Wittchen (2000) found that only 17% of their representative community sample (participants between 14 and 24 years of age) had experienced a traumatic event according to DSM-IV A1 and A2 criteria. Essau, Conradt, and Petermann (2000) reported a prevalence of 22.5% in their 12- to17-years-old representative sample. However, when comparing the prevalence rates of traumatic events in the present study with those in the latest epidemiological study in Switzerland, the results are comparable. Landolt, Schnyder, Maier, Schoenbucher, and Mohler-Kuo (2013) found that 56.1% of the adolescents in their representative sample reported at least one traumatic event on the UCLA-PTSD-RI. The relatively higher prevalence of traumatic events reported by Landolt et al. (2013) and in the present study might be explained by a methodological issue: whereas Perkonigg et al. (2000) and Essau et al. (2000) used structured interviews to assess trauma history, both the present study and Landolt et al. (2013) used self-report measures. At the same time, this difference could be explained by a cohort effect, for example, an increase in trauma rates over the past 15 years. Thus, the question of the extent to which the NS in the present study is representative of the prevalence of traumatic events might depend on both the methodology of the studies and a possible increase in rates. However, because there are no actual epidemiological studies investigating the trauma rates in Germany using structured interviews, this aspect cannot be clarified sufficiently.

## Conclusions

We conclude that the German TSC-C is a reliable instrument for the assessment of trauma-related symptoms on six different scales in adolescents between the ages of 13 and 21. We found evidence that the TSC-C scales measure the aspects for which they were designed. It is useful for German researchers to be able to compare the German results of studies on trauma symptoms in children and adolescents with international results. Furthermore, it is useful for helping German clinicians provide individual trauma symptoms profiles, which are needed to plan appropriate therapeutic help for traumatized children. Further investigations should study the psychometric properties of the German TSC-C in children younger than 13 years of age.

## Supplementary Material

Reliability, factor structure, and validity of the German version of the Trauma Symptom Checklist for Children in a sample of adolescents

Reliability, factor structure, and validity of the German version of the Trauma Symptom Checklist for Children in a sample of adolescents
