# Supplementary material for: Reliability, factor structure, and validity of the German version of the Trauma Symptom Checklist for Children in a sample of adolescents
Source: Eur J Psychotraumatol. 2015 Oct 23;6:10.3402/ejpt.v6.27966. doi: 10.3402/ejpt.v6.27966 (PMC4620686; doi:10.3402/ejpt.v6.27966)
Supplement: Reliability, factor structure, and validity of the German version of the Trauma Symptom Checklist for Children in a sample of adolescents [file EJPT-6-27966-s001.doc]

Supplementary File 1

Detailed information for studies on TSC-C

| **Study** | **Setting** | **Country/region** | **Sample size** | **Trauma history** | **Age range** |
| --- | --- | --- | --- | --- | --- |
| Crouch, Smith, Ezzell, & Saunders, 1999 | Patients who presented themselves for inpatient or outpatient clinical services | USA | *N* = 80 | Sexual abuse (100%) | Range: 8 - 17; *M* = 12.76; *SD* = 2.7 |
| Lanktree et al., 2008 | Clients at one of two child abuse treatment centers who were assessed for maltreatment exposure and psychological symptoms as part of their normal intake evaluation | USA | *N* = 310 | Sexual abuse (49.4%)  Physical abuse (20.6%)  Witnessing domestic violence (41.9%) | Range: 8 - 12; *M* = 9.7; *SD* = 1.5 |
| Sadowski & Friedrich, 2000 | Patients recruited from a child and adolescent psychiatry inpatient unit | USA, Mayo Clinic | *N* = 119 | Physical abuse (24.5%)  Sexual abuse (26.9%)  Physical neglect (7.1%) | Range: 13 - 17; *M* = 15.2; *SD* = 1.3 |

Supplementary File 1

*Continued*

| **Study** | **Setting** | **Country/region** | **Sample size** | **Trauma history** | **Age range** |
| --- | --- | --- | --- | --- | --- |
| Singer, Anglin, Song, & Lunghofer, 1995 | Survey design using an anonymous self-report questionnaire administered to students (grades 9 through 12) in six public high schools during the 1992-1993 school year | USA | *N* = 3735 | Part of the sample had experienced physical violence and/or sexual abuse/assault as either victims or witnesses | Range: 14 – 19; *M* = 16; *SD* = 1.2 |
| Mohammadkhani, Nazari, Dogaheh, Mohammadi, & Azadmehr, 2007 | 1. Normative sample: school children from junior high schools in Tehran 2. Clinical sample: runaway children | Iran | a) *N* = 3042  b) *N* = 141 | No information | 1. Range: 11 - 16; *M* = 13.09; *SD* = .94 2. Range: 11 - 16; *M* = 13.98; *SD* = 1.58 |

Supplementary File 1

*Continued*

| **Study** | **Setting** | **Country/region** | **sample size** | **trauma history** | **age range** |
| --- | --- | --- | --- | --- | --- |
| Nilsson, Wadsby & Svedin, 2008 | 1. Normative sample: investigation in schools 2. Clinical group: patients from a child and adolescent psychiatric outpatient clinic | Sweden | 1. *N* = 728 2. *N* = 91 | 1. No information 2. Sexual abuse (100%) | 1. Range: 10 - 17; *M* = 13.2; *SD* = 1.8 2. Range: 10 - 17; boys: *M* = 12.3; *SD* = 2.4; girls: *M* = 13.9; *SD* = 2.2 |
| Li et al., 2009 | Children affected by HIV/AIDS in China (orphaned by HIV/ AIDS or living with HIV-infected parents) | China | *N* = 1221  (296 double orphans, 459 single orphans, 466 children with HIV-infected parents) | Mean of 4.32 traumatic events (no further information); parents had died or were severely ill | Range: 6-18;  *M* = 12.86  *SD* = 2.25 |

Supplementary File 1

*Continued*

| **Study** | **Setting** | **Country/region** | **sample size** | **trauma history** | **age range** |
| --- | --- | --- | --- | --- | --- |
| Chung, 2014 | 1. Normative sample: school children 2. Traumatized group: patients recruited from the Sunflower Center for the treatment of sexually abused children in Daegu | Korea | 1. *N* = 405 2. *N* = 73 | 1. No information 2. Sexual abuse (100%) | 1. Range: 10-16; boys: *M* = 12.0; *SD* = 2.1, girls: *M* = 11.61; *SD* = 2.4 2. Range: 8 - 17; boys: *M* = 10.1; *SD* = 1.3; girls: *M* = 10.3; *SD* = 2.6 |
