# Supplementary material for: Reliability, factor structure, and validity of the German version of the Trauma Symptom Checklist for Children in a sample of adolescents
Source: Eur J Psychotraumatol. 2015 Oct 23;6:10.3402/ejpt.v6.27966. doi: 10.3402/ejpt.v6.27966 (PMC4620686; doi:10.3402/ejpt.v6.27966)
Supplement: Reliability, factor structure, and validity of the German version of the Trauma Symptom Checklist for Children in a sample of adolescents [file EJPT-6-27966-s002.doc]

Supplementary file 2

Table a

*Correlations between the TSC-C clinical scales and additional* questionnaires in the normative group (N = 583)

| **Clinical scale** | **CES-D** | **STADI** | **UCLA-PTSD-RI** |
| --- | --- | --- | --- |
| **TSC-C ANG** | .53** | .48** | .54** |
| **TSC-C ANX** | .64** | .60** | .63** |
| **TSC-C DEP** | .72** | .56** | .62** |
| **TSC-C DIS** | .59** | .53** | .61** |
| **TSC-C PTS** | .63** | .53** | .76** |
| **TSC-C SC** | .18** | .20** | .27** |

*Note*. ANG = Anger; ANX = Anxiety; DEP = Depression; DIS = Dissociation; PTS = Posttraumatic Stress; SC = Sexual Concerns; * *p* < .05; ** *p* < .01; ns = not significant.

Table b

*Correlations between the TSC-C clinical scales and additional* questionnaires in the clinical group (N = 41)

| **Clinical scale** | **A-DES** | **Depression score** | **UCLA-PTSD-RI** |
| --- | --- | --- | --- |
| **TSC-C ANG** | .57** | .63** | .53** |
| **TSC-C ANX** | .43** | .57** | .67** |
| **TSC-C DEP** | .48** | .81** | .75** |
| **TSC-C DIS** | .71** | .76** | .60** |
| **TSC-C PTS** | .40** | .52** | .80** |
| **TSC-C SC** | .22 ns | .33* | .31* |

*Note*. ANG = Anger; ANX = Anxiety; DEP = Depression; DIS = Dissociation; PTS = Posttraumatic Stress; SC = Sexual Concerns; * *p* < .05; ** *p* < .01; ns = Not significant.
